# Defeat, Entrapment, and Positive Future Thinking: Examining Key Theoretical Predictors of Suicidal Ideation Among Adolescents

**DOI:** 10.3389/fpsyg.2021.590388

**Published:** 2021-03-04

**Authors:** Olivia H. Pollak, Eleonora M. Guzmán, Ki Eun Shin, Christine B. Cha

**Affiliations:** Department of Clinical and Counseling Psychology, Teachers College, Columbia University, New York, NY, United States

**Keywords:** suicide, defeat, entrapment, future thinking, integrated motivational-volitional model, adolescence

## Abstract

Adult-based suicide theories have determined much of what we know about suicidal ideation. Here, we investigate the extent to which elements of the *Integrated Motivational-Volitional* (IMV) model generalize to adolescence, a period when rates of suicidal ideation increase dramatically. In a sample of community-based adolescents (*n* = 74), we tested whether defeat and entrapment related to suicidal ideation, and whether poor positive future thinking abilities exacerbated this association. Consistent with the IMV model, we found that defeat/entrapment was associated specifically with history of suicidal ideation, and not with history of suicide attempt. Defeat/entrapment was related to baseline suicidal ideation severity above and beyond depressive symptoms. While defeat/entrapment predicted future suicidal ideation controlling for history of ideation, it did not do so controlling for depressive symptoms. Counter to the IMV model, we initially found that the association between defeat/entrapment and suicidal ideation was strongest among adolescents with *greater* positive future thinking abilities. This was driven by the tendency to imagine more positive future events, particularly those that are less realistic and achievable. These findings call for a more nuanced understanding of defeat/entrapment and positive future thinking among adolescents, particularly in how they interact to predict recurrent suicidal ideation.

## Introduction

Approximately 16–18% of adolescents report experiencing suicidal ideation each year (Centers for Disease Control and Prevention, National Center for Health Statistics, 2015, Ivey-Stephenson et al., 2020), and approximately one-third of suicidal adolescents go on to attempt suicide (Nock et al., 2013). Despite the prevalence and severity of these outcomes, our understanding of why suicidal thoughts and behaviors (STBs) emerge and persist during adolescence is limited. One cited reason for this is that suicide research to date has largely examined the same narrow set of risk factors—most of which show small effect sizes for prediction of STBs (Franklin et al., 2017). Further, adult samples account for a majority of the risk factor literature over the past 50 years (Franklin et al., 2017). This discrepancy is puzzling, given that rates of suicidal ideation escalate dramatically between the ages of 12 and 17 (Nock et al., 2008, 2013), and suicidal thoughts may transition quickly to behaviors among this age group (Glenn et al., 2017a). Adolescence represents a high-risk period for onset of STBs, yet these outcomes are notably understudied in this population.

In addition to a relative lack of empirical work on adolescence compared to adulthood, there currently exist no adolescent-specific theories of suicide. In the past decade, researchers have posited several theories to explain development of suicidal ideation, and who will transition from suicidal thoughts to action (e.g., *Interpersonal Psychological Theory of Suicide*; Joiner, 2005; Van Orden et al., 2010; *Three-Step Theory*; Klonsky and May, 2015). However, these and most suicide theories are age-agnostic, or else allude to—rather than center around—developmental considerations germane to adolescence. Further, leading theories are infrequently tested among youth. For example, a 2017 meta-analysis of research on the Interpersonal Psychological Theory of Suicide found that fewer than 5% of studies were conducted among youth under 18 years (Chu et al., 2017). There is a need to test the extent to which prevailing theories generalize to adolescence, and if needed, pursue more developmentally sensitive explanations for suicidal ideation earlier in life.

Among existing suicide theories, the *Integrated Motivational-Volitional* model (IMV; O’Connor, 2011) may be a particularly promising framework to explain suicidal ideation among adolescents. The IMV not only offers one of the most detailed explanations for the emergence of suicidal ideation but also incorporates constructs that may be especially relevant to adolescence. The IMV adopts an “ideation-to-action” framework to explain development of suicidal ideation, and the transition from suicidal thoughts to behaviors. It posits that experiences of *defeat* (i.e., failed social struggle and feelings of being brought down), triggered by stressful life events or other environmental precipitants, lead to *entrapment* (i.e., perceived inability to escape or be rescued from aversive situations)—and ultimately suicidal ideation (O’Connor and Kirtley, 2018). Indeed, defeat and entrapment have been linked with suicidal ideation in some prior work (for overviews, see O’Connor and Kirtley, 2018; O’Connor and Portzky, 2018); however, most of these studies have involved adult samples (e.g., O’Connor et al., 2013; Owen et al., 2018). Empirical studies involving youth are limited and have yielded mixed findings. In one of the few studies among adolescents, entrapment was associated cross-sectionally with suicidal ideation (Park et al., 2010), and one prior study with young adults showed mixed findings, suggesting that defeat but not entrapment predicts future suicidal ideation (Taylor et al., 2011). The impact of defeat and entrapment on suicidal ideation warrants clarification, as well as further replication, in younger populations.

A critically understudied component of the IMV model is moderators that may either enhance or mitigate the effects of defeat and entrapment on suicidal ideation. Among the moderators proposed by the IMV model, *positive future thinking* is an especially promising cognitive process that may mitigate risk for suicidal ideation. Moderators such as positive future thinking, or the ability to imagine desirable events that may occur in one’s life, can help mitigate “setting conditions” for transitioning into suicidal thoughts and behaviors (O’Connor, 2011). Adult-based studies suggest the potential importance of positive future thinking in relation to suicidal ideation: distinguishing it from negative future thinking in suicidal individuals (MacLeod et al., 1993, 1997, 1998, 2005; Hunter and O’Connor, 2003), and demonstrating its prediction of suicidal ideation above and beyond hopelessness^1^ (O’Connor et al., 2008). Despite these intriguing theoretical bases, no studies to our knowledge have examined associations between positive future thinking, defeat, and entrapment in predicting future suicidal ideation. Moreover, work exploring future thinking and suicidal ideation (i.e., independent of defeat and entrapment) has been largely limited to adult samples.

It is especially important to explore future thinking in adolescence for two reasons. First, there is a notable improvement in this cognitive ability during this developmental period. Numerous studies suggest that children and adolescents become more oriented toward the future, rather than the present, across development (e.g., Steinberg et al., 2009). Adolescents in particular, relative to children, have been shown to provide more episodic and semantic details when generating future events (Gott and Lah, 2014); this may help prepare them for key developmental tasks of adolescence into early adulthood, including formulation of values, identity, and goals (Marcia, 1980; Nurmi, 1991). Second, future-oriented cognitions have been shown to moderate the association between other psychological traits (e.g., impulsivity) and self-harming behaviors in adolescents (e.g., Chen and Vazsonyi, 2011). Future thinking thereby shows promise as a way to modulate risk for self-injurious thoughts and behaviors, potentially extending to suicidal ideation as the IMV model would predict. Given the developmental salience of future thinking, there is reason to hypothesize that this cognitive process may play a role in modulating risk for suicidal ideation among adolescents, specifically.

Building on prior work, the present study marks the first investigation of how defeat/entrapment, positive future thinking, and their interaction may prospectively predict suicidal ideation during adolescence. We explored the combined construct of *defeat/entrapment* in light of more recent findings suggesting that defeat and entrapment are best captured as a single factor (Griffiths et al., 2015). Specifically, we pursued two aims. First, we aimed to test the strength and specificity of the proposed defeat/entrapment-to-suicidal ideation pathway in adolescents, among whom empirical tests of this association—and suicide theory generally—are lacking. In pursuit of this aim, we directly tested cross-sectional and prospective associations between defeat/entrapment and suicidal ideation among adolescents, and between nonsuicidal and suicidal adolescents. Specifically, we tested whether: (1) defeat/entrapment predicts suicidal ideation cross-sectionally; (2) defeat/entrapment distinguishes between adolescents along the continuum of STBs (i.e., suicidal ideation vs. suicide attempt); and (3) defeat/entrapment prospectively predicts suicidal ideation at two follow-up time points (i.e., 3 and 6 months). We hypothesized that greater defeat/entrapment would distinguish suicidal ideation from no suicidal ideation history, but would not distinguish suicidal ideation history from suicide attempt history. We further hypothesized that greater defeat/entrapment would correspond with greater suicidal ideation at baseline, as well as 3- and 6-months later. Second, we aimed to explore how poor future thinking abilities may alter the association between defeat/entrapment and suicidal ideation. In pursuit of our second aim, we tested whether positive future thinking moderates the association between defeat/entrapment and suicidal ideation. Given prior work linking deficits in *positive* future thinking and suicidal ideation, we hypothesized that greater positive future thinking abilities would mitigate the association between defeat/entrapment and suicidal ideation.

## Materials and Methods

### Participants

Participants were adolescents (*n* = 74) recruited from the community to participate in a larger study examining cognitive deficits in suicidal adolescents. Participants ranged from 12 to 19 years (*M* = 16.27, *SD* = 2.21) and were racially diverse (25.7% White; 21.6% Black; 21.6% Asian; 29.7% other; and 1.4% unknown) and majority non-Hispanic (70.3%; 29.7% Hispanic).

The study recruited adolescents with a past-year history of suicidal ideation, as well as adolescents who had never experienced suicidal thoughts or behaviors. Across the final sample included in this investigation, 41.9% (*n* = 31) of participants endorsed history of suicidal ideation (i.e., with or without suicide attempt history), and 10.8% (*n* = 8) endorsed history of suicide attempt. Of note, we distinguish between “history of ideation” and “history of ideation *only*.” In the following sections, “history of ideation” refers to adolescents with *any* history of suicidal ideation, who may or may not also have a history of suicide attempt. However, “history of ideation only” refers to adolescents with a history of suicidal ideation but not suicide attempt. These distinctions are especially pertinent to Aim 1 data analyses and results, described below.

### Measures

#### Short Defeat and Entrapment Scale

The Short Defeat and Entrapment Scale (SDES; Griffiths et al., 2015) is an 8-item self-report measure assessing feelings of defeat and entrapment over the past week. Participants indicate the extent to which they identify with eight statements on a 5-point Likert scale (i.e., 0 = *Not at all like me* to 4 = *Extremely like me*). Items assessing defeat include “I feel defeated by life” and “I feel that there is no fight left in me,” while those assessing entrapment include “I can see no way out of my current situation” and “I would like to escape from my thoughts and feelings.” The SDES has demonstrated excellent internal consistency (Griffiths et al., 2015).

#### Suicidal Ideation Questionnaire

The Suicidal Ideation Questionnaire (SIQ; Reynolds, 1988) is a 30-item self-report measure assessing frequency of suicidal thoughts over the past month. Items are scored on a 7-point Likert scale (i.e., 0 = *I never had this thought* to 6 = *Almost every day*) and assess frequency of both passive (e.g., “I thought about death”) and active (e.g., “I thought about how I would kill myself”) suicidal thoughts. The SIQ has been shown to have very strong psychometric properties (Reynolds, 1988).

#### Self-Injurious Thoughts and Behaviors Interview-Revised

The Self-Injurious Thoughts and Behaviors Interview-Revised (SITBI-R; Fox et al., 2020) is a semi-structured interview assessing presence and frequency of suicidal and nonsuicidal thoughts and behaviors across various time frames (e.g., lifetime, past year, past week, etc.). This investigation relied on participants’ answers to two questions on the SITBI-R: one question assessing lifetime history of suicidal ideation (i.e., “Have you ever had thoughts of killing yourself?”) and one question assessing lifetime history of suicide attempts (i.e., “Have you ever tried to kill yourself?”). This has been validated in adolescents, and modules for suicidal ideation and attempt reveal perfect inter-rater reliability for lifetime presence of suicidal ideation and attempt, as well as excellent convergent validity with the SIQ (Fox et al., 2020; Gratch et al., in press).

#### Quick Inventory of Depressive Symptomatology-Self Report

The Quick Inventory of Depressive Symptomatology-Self Report (QIDS-SR; Rush et al., 2003) is a 16-item self-report measure assessing depressive symptoms aligned with the nine symptom criteria domains of Major Depressive Disorder, including sad mood, sleep disturbance, and changes in appetite and weight. The QIDS-SR has been shown to have strong psychometric properties, including concurrent validity with other measures of depression (Reilly et al., 2015) and reliability when used with adolescents (*α*s ≥ 0.80; Bernstein et al., 2010). In this investigation, total QIDS-SR scores were calculating excluding item 12 (assessing suicidal ideation).

#### Future Thinking Task

The Future Thinking Task (FTT; MacLeod et al., 1998) assesses participants’ ability to generate and list anticipated future events in their lives across distinct future time frames. In this investigation, we assessed three time periods: the next week, next 3 months (i.e., to fit the 3-month follow-up time frame), and next 5–10 years. Participants are asked to separately generate positive and negative events for each future time period, for a total of six sets of events. For each set, participants were specifically instructed to “think of potential events that may occur in your future” within the given time frame and were provided 1 min to speak aloud as many positive events and, in separate sets, negative events as they could. This study examined positive events, defined as “things you are looking forward to that you think you would enjoy if they did occur.” Events could be trivial or important and planned or unplanned, but participants were asked to generate specific, realistic events that might reasonably happen and would last just a few minutes or hours. Additionally, participants rated the emotional valence (i.e., “What are the types of emotions associated with this event?”) and likelihood (i.e., “How likely is it that this event will occur?”) of each event on 5-point Likert scales (i.e., valence: 0 = *Very negative* to 5 = *Very positive*; likelihood: 0 = *Not at all* to 5 = *Extremely*). Interviewers recorded participants’ event descriptions and valence and likelihood ratings. Following conventional FTT scoring procedures (MacLeod et al., 1998, 2005), a composite positive FTT score (i.e., FTT-Pos) was calculated by multiplying the total number of positive events generated across the three positive event sets; the mean valence rating across all positive events; and the mean likelihood rating across all positive events.

### Procedure

Adolescent participants were recruited from New York City and the broader tristate area *via* flyers, community fairs, and online advertisements. After completing a phone screen to determine study eligibility (12–19 years, English proficiency, and no high/imminent suicide risk), participants completed an in-person laboratory visit. Participants under 18 years of age were accompanied by a parent or guardian, who provided informed consent for their child’s participation. Adolescents completed study self-report measures (i.e., SDES, SIQ, and QIDS-SR) privately on a computer. The FTT and SITBI-R were administered by trained interviewers. Adolescent participants were compensated with a $40 Amazon.com gift card. Adolescents were sent follow-up surveys *via* email 3 and 6 months after their lab visit to assess suicidal ideation (i.e., SIQ). At 3-month follow-up, participants were also provided a list of the positive and negative events they had generated in the FTT during the baseline lab visit—specifically, those events generated for the “next three months” time frame set—and were asked to indicate whether the events had actually occurred in the 3 months prior.

#### Data Analyses

Analyses were conducted with the SPSS statistical package (IMB SPSS Statistics, version 25.0). SDES, SIQ (i.e., baseline, 3-month follow-up, and 6-month follow-up), QIDS-SR, and FTT-Pos composite scores were transformed to satisfy assumptions of normality prior to further analyses. Additionally, missing data were observed for follow-up SIQ variables (i.e., 3-month and 6-month). Little’s Missing Completely at Random (MCAR) test was not significant and supported the MCAR assumption, *χ*^2^(18) = 19.21, *p* = 0.38, supporting the handling of missing data *via* pairwise deletion. Further diagnostic analyses using independent samples *t*-tests revealed no significant differences in any study variables (i.e., SDES, baseline SIQ, QIDS, and Pos-FTT) between those who did vs. did not have 3-month SIQ data, *t*(72) = −0.69 to 0.45, *p*s = 0.49–0.71, and 6-month SIQ data, *t*(72) = −1.35 to 1.75, *p*s = 0.09–0.53. There was also no correlation between history of suicidal ideation at baseline and completion of 3- or 6-follow-ups (*χ*^2^ = 0.01–0.03, *p*s = 0.87–0.94), suggesting that adolescents with a history of suicidal ideation were not more or less likely to complete follow-ups than controls.

To test our first aim, we conducted a linear regression testing the cross-sectional association between defeat/entrapment and suicidal ideation, with SDES scores as the independent variable and baseline SIQ scores as the dependent variable. *Post-hoc* analyses also controlled for depressive symptoms (QIDS-SR) as a covariate. Additionally, we compared defeat/entrapment across three mutually exclusive groups: nonsuicidal adolescents (i.e., no history of suicidal ideation or attempt); adolescents with a history of suicidal ideation only (i.e., history of suicidal ideation but not attempt); and adolescents with a history of suicide attempt (i.e., history of suicidal ideation and attempt), using one-way ANOVA. For this analysis, adolescents were classified into the category of STBs reflecting the greatest level of severity endorsed, based on lifetime history of suicidal ideation and suicide attempt(s) assessed in the baseline lab visit using the SITBI-R.

In pursuit of our second aim, we tested the association between defeat/entrapment and suicidal ideation across two follow-up time points (i.e., 3-month and 6-month) *via* multiple linear regression models, with SDES scores as the independent variable and SIQ scores as the dependent variable. Prospective models predicting follow-up SIQ (i.e., at 3- and 6-months) also included baseline SIQ as a covariate. *Post-hoc* analyses also controlled for depressive symptoms (QIDS-SR) as a covariate.

Thirdly, to test positive future thinking as a moderator, defeat/entrapment (i.e., SDES) and positive future thinking (i.e., FTT-Pos) variables were centered and multiplied to create an interaction term. Linear regressions were conducted with SDES, FTT-Pos, and (for analyses predicting follow-up SIQ) baseline SIQ entered in the first step. The interaction term was entered in the second step. *Post-hoc* probing analyses were conducted following guidance on testing moderation (Aiken and West, 1991; Holmbeck, 2002). Results of these *post-hoc* analyses were graphed at low (−1 SD below the mean) and high (+1 SD above the mean) levels of positive future thinking. Similar to Aims 1 and 2, additional *post-hoc* analyses explored baseline depressive symptoms (i.e., QIDS-SR) as a covariate in moderation models that significantly predicted suicidal ideation.

## Results

Descriptive statistics and Pearson’s *r* correlations for SDES, SIQ (i.e., baseline and 3- and 6-month follow-ups), FTT-Pos, and QIDS-SR are presented in Table 1. On average, participants generated between 17 and 18 positive future events across the three FTT positive event sets (*M* = 17.63, *SD* = 5.97, *range*: 6–35). Across participants, positive events tended to be rated as moderately likely to occur (*M* = 3.82, *SD* = 0.55) and fairly positive in valence (*M* = 4.42, *SD* = 0.29). Positive events generated included things such as desired activities (e.g., “go to the Museum of Natural History”); anticipated accomplishments (e.g., “get 100% on vocab test”); receipt of gifts, toys, or other possessions (e.g., “Mom buys me a new game”); and completion of, or relief from, unwanted tasks or responsibilities (e.g., “be done with all my appointments”).

**Table 1:** Bivariate correlations and descriptive statistics.

	1.	2.	3.	4.	5.	6.
1. SDES	--					
2. SIQ (baseline)	0.71^**^	--				
3. SIQ (3-month)	0.64^**^	0.71^**^	--			
4. SIQ (6-month)	0.53^**^	0.50^**^	0.72^**^	--		
5. FTT-Pos	−0.06	−0.16	−0.01	−0.20	--	
6. QIDS-SR	0.77^**^	0.60^**^	0.64^**^	0.59^**^	−0.15	--
Mean	6.96	18.89	24.50	23.71	299.18	7.50
Standard deviation	7.35	24.92	29.64	31.90	119.27	**4.43**

Table presents Pearson’s *r* values. SDES, Short Defeat and Entrapment Scale; SIQ, Suicide Ideation Questionnaire; FTT-Pos, Positive Future Thinking Task (composite score for positive trials); QIDS-SR, Quick Inventory of Depressive Symptoms-Self Report (excludes item 12 assessing suicidal ideation).

** *p* <0.01.

### Aim 1

In linear regression analyses, SDES significantly predicted baseline SIQ scores. SDES remained predictive of baseline SIQ in multiple linear regression models controlling for depressive symptoms (Table 2).

**Table 2:** Cross-sectional and prospective linear regression analyses predicting suicidal ideation by defeat/entrapment and depressive symptoms.

	Model 1			Model 2		
	Baseline SIQ	3-mo SIQ	6-mo SIQ	Baseline SIQ	3-mo SIQ	6-mo SIQ
*R* ^2^	0.50	0.68	0.54	0.51	0.68	0.55
SDES (*β*)	0.70^**^	0.23^*^	0.12	0.58^**^	0.17	0.04
Baseline SIQ (*β*)	--	0.65^**^	0.64^**^	--	0.63^**^	0.62^**^
QIDS-SR (*β*)	--	--	--	0.17	0.08	0.55

SDES, Short Defeat and Entrapment Scale; SIQ, Suicide Ideation Questionnaire; QIDS-SR, Quick Inventory of Depressive Symptoms-Self Report (excludes item 12 assessing suicidal ideation). 3-mo and 6-mo SIQ refer to suicidal ideation severity at 3-month follow-up and 6-month follow-up, respectively. Model 1 includes baseline SIQ as a covariate. Model 2 includes depressive symptoms and baseline SIQ as covariates. R^2^, model explained variance, β, standardized beta.

* *p* < 0.05;

** *p* < 0.01.

**Figure 1:**
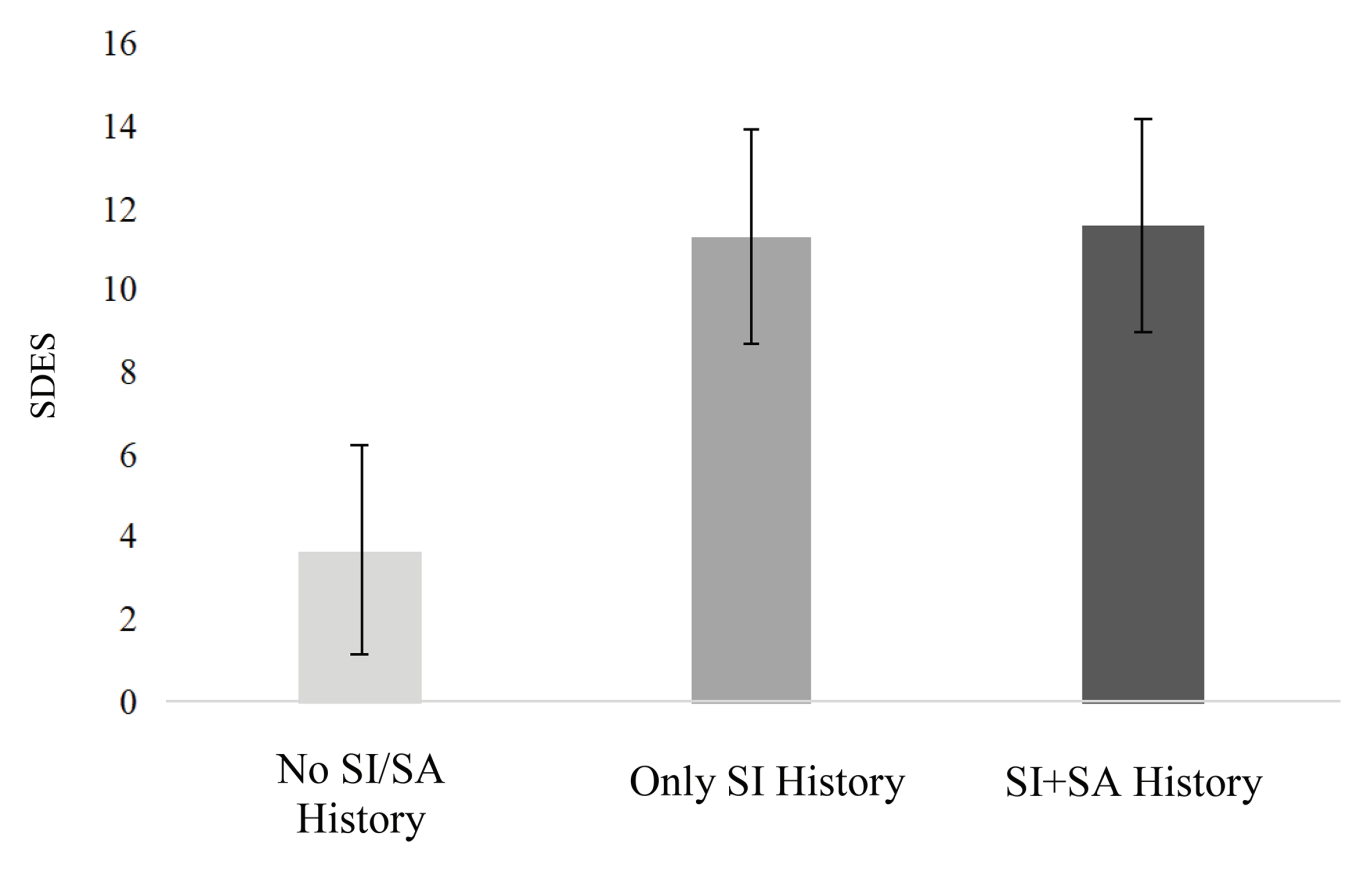
Differences in defeat/entrapment across suicidal and nonsuicidal adolescents. SDES, Short Defeat and Entrapment Scale; No SI/SA History, no history of suicidal ideation or attempt; Only SI History, history of suicidal ideation but not attempt; SI+SA History, history of suicidal ideation and suicide attempt. Error bars represent standard error.

Short Defeat and Entrapment Scale scores significantly differed across groups, *F*(2, 71) = 14.34, *p* < 0.001 (Figure 1). Specifically, adolescents with a history of ideation only endorsed greater feelings of defeat/entrapment (*M* = 11.35, *SD* = 7.67) compared to nonsuicidal adolescents (*M* = 3.74, *SD* = 5.29; *p* < 0.001, *d* = 1.16). There was no difference in defeat/entrapment between adolescents with a history of ideation only and those with a history of attempts (*M* = 11.63, *SD* = 7.65; *p* = 1.00, *d* = 0.04).

Additional *post-hoc* analyses explored the defeat and entrapment subscales of the SDES to determine whether these two constructs showed differential associations with suicidal ideation and attempts. Both defeat scores, *F*(2, 71) = 13.69, *p* < 0.001, and entrapment scores, *F*(2, 71) = 12.06, *p* < 0.001, significantly differed across groups. Group differences mirrored those for SDES total scores. Adolescents with a history of ideation only endorsed significantly higher defeat (*M* = 4.52, *SD* = 4.02) and entrapment (*M* = 6.83, *SD* = 4.24) scores compared to nonsuicidal adolescents (*M*s = 1.23–2.51, *SD*s = 2.73–2.93; *p*s < 0.001, *d*s = 1.16–1.25), even after applying Bonferroni corrections to adjust for multiple comparisons. Neither defeat nor entrapment scores differed between adolescents with a history of ideation only and those with a history of attempts (*p*s = 1.00, *d*s = 0.02–0.03).

### Aim 2

Baseline SDES predicted 3-month SIQ in prospective models controlling for baseline SIQ (Table 2, Model 1). SDES did not predict 6-month SIQ in prospective models controlling for baseline SIQ. After controlling for baseline depressive symptoms, SDES was no longer predictive of 3- or 6-month SIQ (Table 2, Model 2).

### Aim 3

**Figure 2:**
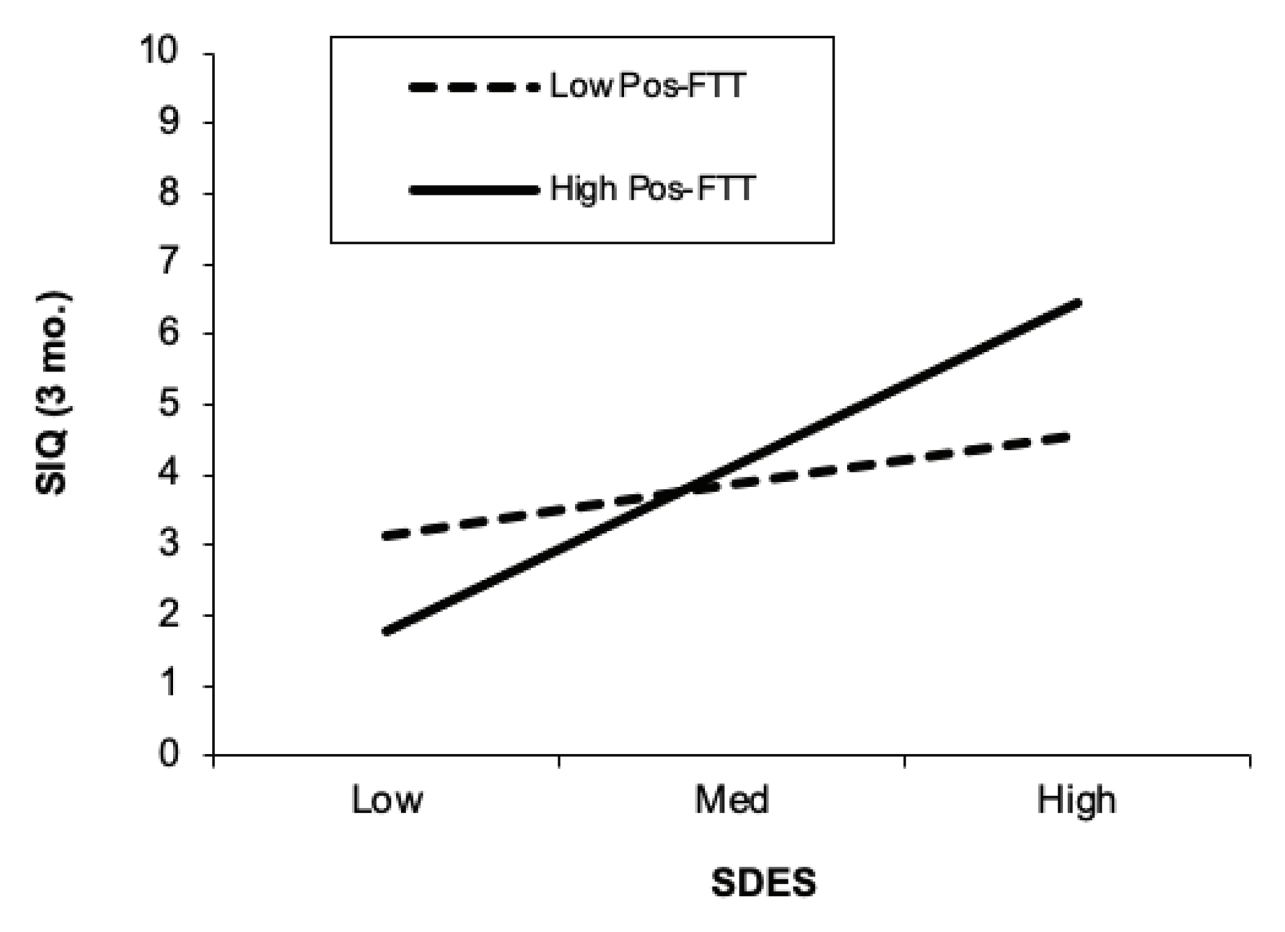
Positive future thinking moderates the association between defeat/entrapment and future (3-month) suicidal ideation. SDES, Short Defeat and Entrapment Scale; SIQ, Suicide Ideation Questionnaire. Greater and lower levels of positive future thinking were defined as +1 SD and −1 SD, respectively. The SIQ scale reflects values of the transformed variable, and not raw scores.

Positive future thinking (FTT-Pos) did not moderate the association between defeat/entrapment and SIQ scores from baseline or the 6-month follow-up (*β*s = −0.01 to 0.03, *p*s = 0.75–0.96). The interaction between defeat/entrapment and FTT-Pos was, however, significant for prediction of 3-month follow-up SIQ (*β* = 0.17, *p* = 0.04). Contrary to hypothesis, results showed that defeat/entrapment was associated with 3-month SIQ among those with *greater* positive future thinking abilities (*β* = 0.36, *p* = 0.02), but not among those with lower positive future thinking abilities (*β* = 0.11, *p* = 0.37; Figure 2). The interaction between defeat/entrapment and FTT-Pos predicted 3-month SIQ at a marginally significant level after controlling for depressive symptoms (*β* = 0.17, *p* = 0.051).

*Post-hoc* analyses further explored elements of FTT-Pos scores to understand exactly which feature of positive future thinking accounted for this significant interaction. We repeated moderation analyses predicting 3-month SIQ using, in separate models, the three values comprising the composite FTT-Pos score: total number of positive events generated across the FTT, mean likelihood of positive events, and mean valence of positive events. The interaction of SDES with total number, but not mean likelihood (*β* = −0.01, *p* = 0.86) or mean valence (*β* = −0.03, *p* = 0.73), of positive future events significantly predicted 3-month follow-up SIQ (*β* = 0.20, *p* = 0.01). Specifically, defeat/entrapment predicted 3-month follow-up SIQ among those who generated more, but not fewer, positive future events (*β* = 0.41 *p* = 0.003). The interaction term remained significant after controlling for depressive symptoms (*β* = 0.19, *p* = 0.02).

**Figure 3:**
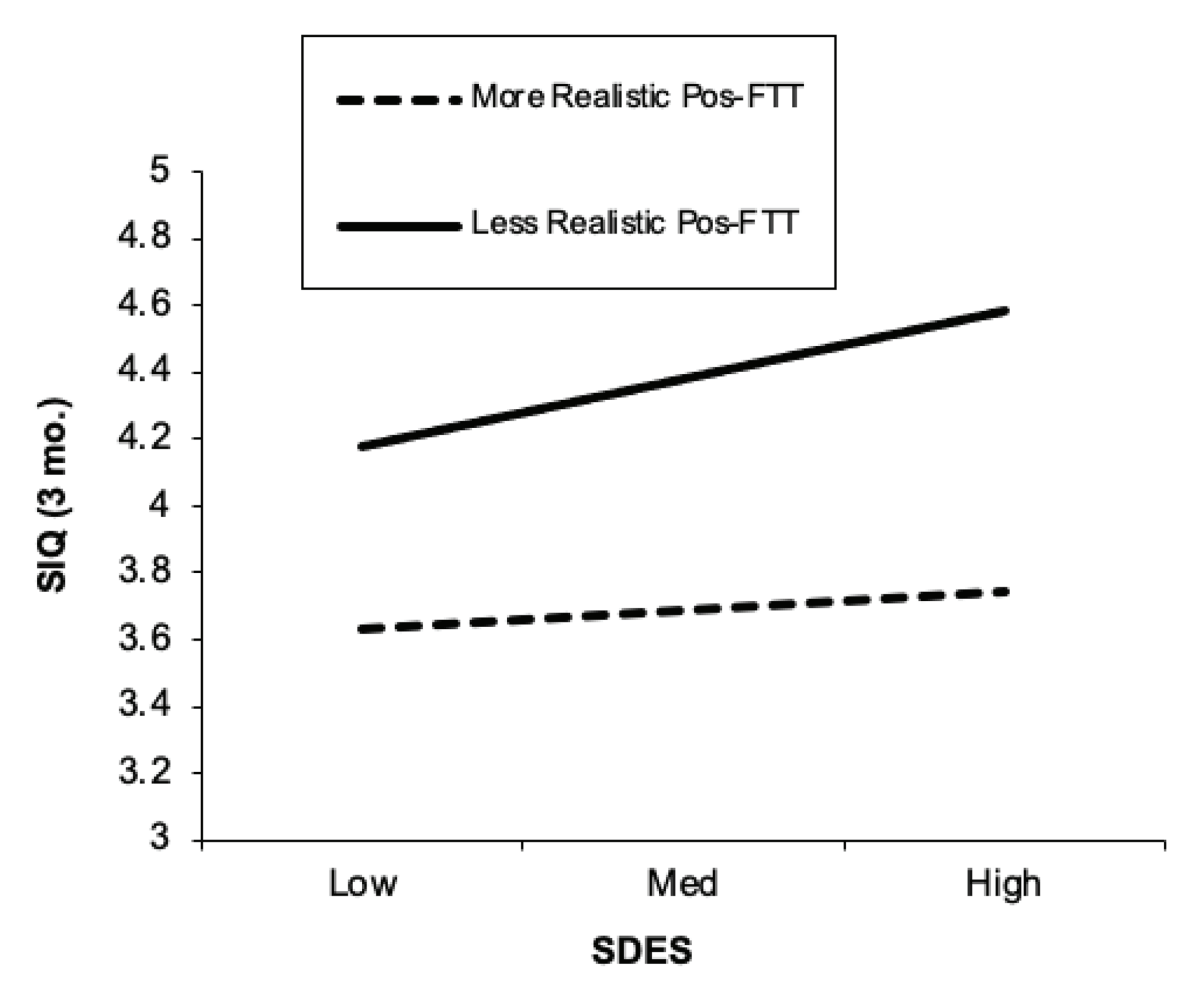
Degree of realistic positive future thinking moderates the association between defeat/entrapment and future (3-month) suicidal ideation. SDES, Short Defeat and Entrapment Scale; SIQ, Suicide Ideation Questionnaire. More vs. less realistic positive future thinking levels (i.e., less unrealistic vs. more unrealistic) were defined as −1 SD and +1 SD, respectively. The SIQ scale reflects values of the transformed variable, and not raw scores.

We conducted additional *post-hoc* analyses addressing how positive future thinking may have been maladaptive in nature. Imagining many positive future events that are, for instance, detached from reality and unlikely to occur would presumably not be helpful. To determine how realistic adolescents’ imagined positive events were, we assessed whether those events listed from baseline occurred over the next 3 months and calculated what proportion of them did *not* occur (i.e., unrealistic positive future thinking index). Indeed, the proportion of unrealistic positive future thinking moderated the association between defeat/entrapment and suicidal ideation 3 months later (*β* = 0.17, *p* = 0.03). We probed this result at higher (+1 SD above the mean) and lower (−1 SD below the mean) levels of unrealistic future thinking (i.e., proportion of unrealized positive events) and found that defeat/entrapment predicted 3-month SIQ among those with less realistic future thinking (i.e., higher proportions of unrealized positive events; *β* = 0.42, *p* = 0.004), but not among those with more realistic future thinking (i.e., lower proportions of unrealized positive events; *β* = 0.12, *p* = 0.38; Figure 3). The interaction term between defeat/entrapment and unrealistic positive future thinking remained significant after controlling for depressive symptoms (*β* = 0.16, *p* = 0.045).

## Discussion

This investigation yielded three main findings. First, defeat/entrapment was associated with history of suicidal ideation, as demonstrated by a significant cross-sectional association between defeat/entrapment and ideation severity, and differences in defeat/entrapment between adolescents with and without a history of suicidal ideation. Moreover, this association was not accounted for by depressive symptoms. These cross-sectional findings align with the IMV model’s emphasis on the role of defeat/entrapment in explaining suicidal ideation (vs. suicide attempt), and shows that such associations exist in adolescence. Consistent with the IMV model, defeat/entrapment was specific to history of suicidal ideation: defeat/entrapment distinguished adolescents who had considered suicide from those who had not, but did not distinguish adolescents who had considered suicide from those who had attempted suicide. Indeed, adolescents’ experience of defeat/entrapment may help distinguish between absence vs. presence of suicidal thoughts, but may not offer unique predictive validity in distinguishing ideators who have also engaged in suicidal behaviors (i.e., suicide attempts). Additionally, while some studies show differential associations of defeat vs. entrapment with suicidal ideation (e.g., Taylor et al., 2011; O’Connor et al., 2013), we found that ideators showed elevated scores on both defeat and entrapment subscales, suggesting that both constructs may distinguish adolescents who have previously considered suicide from those who have not.

Second, defeat/entrapment was not a robust predictor of future suicidal ideation among adolescents. Defeat/entrapment predicted 3-month follow-up suicidal ideation controlling for baseline ideation, but did not do so controlling for depressive symptoms. This is inconsistent with prior evidence that defeat can predict future suicidal ideation above and beyond depressive symptoms (Taylor et al., 2011). This may be accounted for by the exceptionally strong association between baseline defeat/entrapment and depressive symptoms observed in our sample. Depressive symptoms and defeat/entrapment may reinforce one another; prior work has shown that while defeat/entrapment predicts depressive symptoms, the reverse is also true (Griffiths et al., 2014). This is also in keeping with concepts of “arrested flight” and certain models of depression (e.g., social rank and arrested defenses models; see Carvalho et al., 2013), which link feelings of defeat and entrapment with depressive symptoms (Gilbert and Allan, 1998). Our results may suggest a similar pattern, such that defeat/entrapment increases ideation severity, and suicidal ideation exacerbates feelings of defeat/entrapment.

Third, we found that positive future thinking moderated the association between defeat/entrapment and suicidal ideation at 3-month follow-up. Contrary to hypotheses, greater positive future thinking abilities exacerbated the association between defeat/entrapment and suicidal ideation three months later. When examining components of the positive future thinking score, it was the number of positive events generated, rather than the perceived likelihood or emotional valence of positive events, that drove this moderating effect. These findings initially appear to contradict the IMV model and other research connecting *low* levels of positive future thinking and suicidal ideation (e.g., MacLeod et al., 1997; Hunter and O’Connor, 2003; O’Connor et al., 2004, 2007, 2008). However, this is not the only case of maladaptive positive future thinking: O’Connor et al. (2015) found that high positive intrapersonal future thinking predicted suicidal behaviors among adults. There are several possible explanations for this unexpected pattern. For instance, such a pattern would emerge if adolescents’ positive future thoughts pertained to maladaptive outcomes that would yield negative consequences if they occurred (e.g., risky behaviors). Similarly, suicidal adolescents could experience suicide-related mental imagery when prompted to imagine positive future events (e.g., flash-forwards and day dreams; Holmes et al., 2007; Selby et al., 2007). These possibilities, while interesting, are unlikely, as our cursory review of event content revealed no such thematic patterns. An additional consideration, albeit tentative, is that similar cognitive processes may underlie thoughts of positive future events and thoughts of death or suicide. Other work shows that suicide-related thoughts or mental imagery are future-oriented, often rated as comforting (Holmes et al., 2007; Crane et al., 2012, 2014), and associated with increases in positive affect (Kleiman et al., 2018). That more severely suicidal adolescents may be better at, or more practiced in, engaging in positively valenced, future-oriented mental imagery—in the form of suicidal thoughts or otherwise—presents an intriguing hypothesis. However, this hypothesis is speculative and inconsistent with a majority of prior findings showing weaker positive future thinking among suicidal adults. Nevertheless, future work might explore differences in cognitive processes underlying future-oriented thoughts and mental imagery between suicidal and nonsuicidal adolescents.

Instead, *post-hoc* analyses revealed an alternative explanation: that adolescents’ positive future thoughts may be unrealistic, such that they do not attain anticipated positive events and thereby experience greater entrapment and ultimately suicidal ideation. In support of this “unachievability hypothesis,” the tendency to imagine less realistic positive future events significantly and robustly moderated the effects of defeat/entrapment on suicidal ideation, such that this association was stronger among adolescents with more unrealistic positive future thinking (i.e., greater proportions of unrealized positive events). Taken together, results suggest that positive future thinking, particularly *unrealistic* positive future thinking, may not always be protective. This finding provides nuance to prior literature largely showing associations between *low* levels of positive future thinking and suicidal outcomes. Future work might attend to characteristics of positive future thinking (e.g., thematic content; perceived likelihood vs. actual occurrence) to further understand how, and under what circumstances, positive future thinking mitigates or heightens risk for suicidal ideation.

Our findings should be interpreted in light of several study limitations. First, the present sample featured a small sample size. This would have increased the risk of Type II error in the case of small effects. Additionally, sample size was further limited by missing follow-up data for prospective analyses. While diagnostic analyses revealed no biases in data missingness, further limiting of sample size represents a notable limitation. Second, we did not assess for verbal fluency. Given the 1 min time limit of the FTT, those who generated higher numbers of positive future events may have done so in part because of greater verbal fluency abilities, thus yielding higher positive FTT scores. Future work should control for general cognitive or verbal fluency in order to remove this potential confound. Third, we did not account for the potential effects of mood on FTT performance. One study assessing the effect of mood on positive future thinking found that positive future thinking decreased following a negative mood induction (O’Connor and Williams, 2014). As we assessed positive future thinking only once, adolescents’ FTT performance could have been influenced by mood during the lab visit, and may therefore not accurately measure general future thinking ability across time and mood states. Fourth, our “unrealistic positive future thinking index” may be driven by factors other than the perceived achievability of events listed at baseline. Finally, the present investigation did not test other critical elements of the IMV model, including the transition from suicidal ideation to attempt. Prospective studies featuring larger and more clinically severe samples would be better suited to explore the transition to suicide attempt.

In sum, we have tested elements of the IMV model explaining suicidal ideation and found that not all aspects of the model can be assumed to generalize to adolescence. We encourage future work to carefully consider age differences in theoretical predictors of suicidal ideation in order to better understand this developmental period. We also encourage further examination of future thinking, including different types of positive future thoughts or differences in positive vs. negative future thinking, among suicide researchers. Examining variability in future thinking is aligned with trends in suicide research toward identifying dysfunctional patterns in basic processes, including those normally considered adaptive, that may characterize suicidal individuals (e.g., Glenn et al., 2017b; Millner et al., 2020). Identifying which protective processes to enhance—and how—will help inform future efforts to disrupt patterns of recurrent suicidal ideation.

## Data Availability Statement

The raw data supporting the conclusions of this article will be made available by the authors, without undue reservation.

## Ethics Statement

The studies involving human participants were reviewed and approved by Teachers College, Columbia University IRB. For minors, written informed consent to participate in this study was provided by the participants’ legal guardian/next of kin.

## Author Contributions

OP: conceptualization, formal analysis, and writing — original draft. EG: conceptualization and writing — review and editing. KS: methodology and writing — review and editing. CC: conceptualization, methodology, project administration, and writing — review and editing. All authors contributed to the article and approved the submitted version.

### Conflict of Interest

The authors declare that the research was conducted in the absence of any commercial or financial relationships that could be construed as a potential conflict of interest.

## Footnotes

^1^As a conceptual aside, positive future thinking is distinct from constructs such as hopelessness not only in emotional valence, but also in scope. Positive future thinking captures a more discrete cognitive ability, whereas hopelessness captures a broader attitude or outlook (i.e., hopelessness about the future, self, and the world; Beck et al., 1974) that may not only recruit future thinking but other psychological processes (e.g., problem-solving abilities, fixed mindset, and low self-efficacy; Millner et al., 2020). Indeed, feelings of defeat and entrapment without promise of future relief may render death an appealing option.
