# Corrigendum: Defeat, Entrapment, and Positive Future Thinking: Examining Key Theoretical Predictors of Suicidal Ideation Among Adolescents

**DOI:** 10.3389/fpsyg.2021.741504

**Published:** 2021-09-16

**Authors:** Olivia H. Pollak, Eleonora M. Guzmán, Ki Eun Shin, Christine B. Cha

**Affiliations:** Department of Clinical and Counseling Psychology, Teachers College, Columbia University, New York, NY, United States

**Keywords:** suicide, defeat, entrapment, future thinking, integrated motivational-volitional model, adolescence

In the original article, there were mistakes in the legends for Figures 2 and 3 as published. The figure legends incorrectly stated that greater and lower levels of positive future thinking (Figure 2) were defined at −1 SD and +1 SD, respectively; and that more vs. less realistic positive future thinking levels were defined at +1 SD and −1 SD, respectively (Figure 3). In fact, it was the opposite. Additionally, the text of the original article also incorrectly stated that higher and lower levels of positive future thinking, and higher and lower levels of unrealistic positive future thinking, were probed at −1 SD below and +1 SD above the mean, respectively. In fact, it was the opposite: higher positive and higher unrealistic future thinking were probed at +1 SD above the mean, and lower positive and lower unrealistic future thinking were probed at −1 SD below the mean. The authors apologize for these errors in reporting and state that they do not change the scientific conclusions of the article in any way.

The correct legends appear below.

**Figure 2** Positive future thinking moderates the association between defeat/entrapment and future (3-month) suicidal ideation. SDES, Short Defeat and Entrapment Scale; SIQ, Suicide Ideation Questionnaire. Greater and lower levels of positive future thinking were defined as +1 SD and −1 SD, respectively. The SIQ scale reflects values of the transformed variable, and not raw scores.

**Figure 3** Degree of realistic positive future thinking moderates the association between defeat/entrapment and future (3-month) suicidal ideation. SDES, Short Defeat and Entrapment Scale; SIQ, Suicide Ideation Questionnaire. More vs. less realistic positive future thinking levels (i.e., less unrealistic vs. more unrealistic) were defined as −1 SD and +1 SD, respectively. The SIQ scale reflects values of the transformed variable, and not raw scores.

A correction has been made to *Procedure, Data Analyses, Paragraph 4*. The corrected paragraph is shown below.

Thirdly, to test positive future thinking as a moderator, defeat/entrapment (i.e., SDES) and positive future thinking (i.e., FTT-Pos) variables were centered and multiplied to create an interaction term. Linear regressions were conducted with SDES, FTT-Pos, and (for analyses predicting follow-up SIQ) baseline SIQ entered in the first step. The interaction term was entered in the second step. *Post-hoc* probing analyses were conducted following guidance on testing moderation (Aiken and West, 1991; Holmbeck, 2002). Results of these *post-hoc* analyses were graphed at low (−1 SD below the mean) and high (+1 SD above the mean) levels of positive future thinking. Similar to Aims 1 and 2, additional *post-hoc* analyses explored baseline depressive symptoms (i.e., QIDS-SR) as a covariate in moderation models that significantly predicted suicidal ideation.

A correction has also been made to *Results, Aim 3, Paragraph 3*. The corrected paragraph is shown below.

We conducted additional *post-hoc* analyses addressing how positive future thinking may have been maladaptive in nature. Imagining many positive future events that are, for instance, detached from reality and unlikely to occur would presumably not be helpful. To determine how realistic adolescents' imagined positive events were, we assessed whether those events listed from baseline occurred over the next 3 months and calculated what proportion of them did *not* occur (i.e., unrealistic positive future thinking index). Indeed, the proportion of unrealistic positive future thinking moderated the association between defeat/entrapment and suicidal ideation 3 months later (β = 0.17, *p* = 0.03). We probed this result at higher (+1 SD above the mean) and lower (−1 SD below the mean) levels of unrealistic future thinking (i.e., proportion of unrealized positive events) and found that defeat/entrapment predicted 3-month SIQ among those with less realistic future thinking (i.e., higher proportions of unrealized positive events; β = 0.42, *p* = 0.004), but not among those with more realistic future thinking (i.e., lower proportions of unrealized positive events; β = 0.12, *p* = 0.38; **Figure 3**). The interaction term between defeat/entrapment and unrealistic positive future thinking remained significant after controlling for depressive symptoms (β = 0.16, *p* = 0.045).

The authors reiterate that these errors do not change the scientific conclusions of the article in any way. The original article has been updated.

## Publisher's Note

All claims expressed in this article are solely those of the authors and do not necessarily represent those of their affiliated organizations, or those of the publisher, the editors and the reviewers. Any product that may be evaluated in this article, or claim that may be made by its manufacturer, is not guaranteed or endorsed by the publisher.
